# People With Lowest Physical Functioning Scores Showed Greatest Improvement After Tirzepatide Treatment

**DOI:** 10.1002/oby.70067

**Published:** 2025-11-04

**Authors:** Xuan Li, Dachuang Cao, Helene Sapin, Fangyu Wang, Theresa Hunter Gibble, Nedina Kalezic Raibulet, Max Denning, Lee M. Kaplan

**Affiliations:** ^1^ Eli Lilly and Company Indianapolis Indiana USA; ^2^ Geisel School of Medicine at Dartmouth and Dartmouth Health Hanover New Hampshire USA

**Keywords:** health‐related quality of life, obesity, patient‐reported outcomes, physical function, tirzepatide

## Abstract

**Objective:**

This post hoc analysis of SURMOUNT trials assessed the association of baseline physical function (PF) with obesity‐related complications (ORCs), efficacy measures, and PF. The mechanism of tirzepatide‐led improvements in PF was evaluated.

**Methods:**

Outcomes were assessed among participants (SURMOUNT‐1 = 2539; SURMOUNT‐3 = 579; SURMOUNT‐4 = 670) grouped by baseline quartiles (Q) of SF‐36v2 PF scores within study (higher scores = better PF). Least‐squares mean changes from baseline in efficacy measures and PF were estimated using ANCOVA. Pearson's correlation between weight reduction and improvement in PF was calculated.

**Results:**

In SURMOUNT‐1, participants with lower baseline PF had more ORCs. Tirzepatide‐treated participants showed similar reductions in weight (kg; −20.1% to −22.8%), waist circumference (−17.2 to −20.2 cm), and BMI (−7.2 to −9.0 kg/m^2^) across quartiles. Participants with lower baseline PF reported greater improvements in PF with tirzepatide (Q1 = 12.5; Q4 = −0.8). Results were similar in SURMOUNT‐3 and SURMOUNT‐4. A weak to mild correlation was noted between weight reduction and improved PF; the strength of correlation decreased from Q1 to Q4.

**Conclusions:**

Lower baseline PF was associated with a higher prevalence of ORCs. Patients taking tirzepatide experienced substantial weight loss, regardless of their baseline PF. Tirzepatide may improve PF through both weight loss‐dependent and ‐independent mechanisms, especially in those with lower baseline PF.

**Trial Registration:**

ClinicalTrials.gov identifiers: SURMOUNT‐1, NCT04184622; SURMOUNT‐3, NCT04657016; SURMOUNT‐4, NCT04660643

**Study Importance**

- What is already known?
  ○ In phase 3 SURMOUNT‐1, ‐3, and ‐4 trials in participants with overweight and obesity without diabetes, treatment with tirzepatide versus placebo led to significant weight reduction and improvements in self‐reported physical and psychological functioning.
- What does this study add?
  ○ The study found that lower baseline physical functioning scores (Q1 = lowest quartile; Q4 = highest quartile) were associated with a higher prevalence of obesity‐related complications. Reductions in body weight, waist circumference, and BMI with tirzepatide were consistent across baseline quartiles (Q1–Q4) of physical functioning scores. Notably, participants with lower baseline physical functioning scores demonstrated greater improvements in physical functioning after tirzepatide treatment.
- How might these results change the direction of research or the focus of clinical practice?
  ○ Limitations in physical functioning do not interfere with weight loss, suggesting that such limitations should not be a contraindication for using tirzepatide.
  ○ Physical functioning improved the most in tirzepatide users with the greatest impairment at baseline. These results may inform treatment decisions, particularly when improving physical functioning is a key treatment goal.
  ○ Our findings suggest that tirzepatide may improve physical functioning through both physiologically induced and weight loss‐dependent mechanisms.

## 1 Introduction

Obesity affected 1 in 8 individuals globally in 2022 and the prevalence of obesity in the US was 40.3% in 2021–2023 [1, 2]. Obesity is associated with an elevated risk of various comorbid conditions, including metabolic, cardiovascular and musculoskeletal diseases, and cancers [2, 3, 4]. Individuals living with obesity often experience limitations in physical function, which negatively affects their ability to perform daily activities, reduces work productivity, and impairs social engagement [4, 5, 6]. Furthermore, weight bias and stigma negatively affect mental health, resulting in an overall decline in health‐related quality of life (HRQoL) [7, 8].

A 5% to ≥ 15% body weight reduction can improve obesity‐related complications (ORCs) and HRQoL, including physical function and psychosocial well‐being, in individuals with obesity [9]. Lifestyle interventions such as diet, physical activity, and behavioral changes are common approaches to manage obesity [10]. However, these approaches can be challenging for individuals with limitations in physical function and can be difficult to sustain over time [10]. Treatment with obesity management medications along with lifestyle interventions is recommended for achieving sustained weight reduction and improving quality of life [10, 11].

Tirzepatide is a once‐weekly glucose‐dependent insulinotropic polypeptide and glucagon‐like peptide‐1 receptor agonist. It has been approved in the US for the treatment of type 2 diabetes (T2D), obesity, and most recently for moderate‐to‐severe obstructive sleep apnea (OSA) [12, 13, 14]. Treatment with tirzepatide resulted in significant weight reduction and improved HRQoL compared to placebo in adults with obesity or overweight without diabetes in phase 3 SURMOUNT‐1 (NCT04184622), SURMOUNT‐3 (NCT04657016), and SURMOUNT‐4 (NCT04660643) clinical trials [15, 16, 17, 18].

While there is evidence of improved physical function with tirzepatide treatment in SURMOUNT trials [18, 19, 20, 21], it remains unclear if the degree of improvement in physical function is consistent across patients with varying baseline levels of physical function. The current post hoc analyses of the SURMOUNT‐1, ‐3, and ‐4 trials aimed to address the following questions: (1) Are duration of obesity and ORCs associated with lower physical function? (2) Are improvements in efficacy measures and patient‐reported outcomes (PROs) with tirzepatide determined by baseline physical function? (3) Does tirzepatide improve physical function through a combination of weight loss‐dependent and weight loss‐independent mechanisms? Answers to these questions can provide valuable insights into the mechanisms of physical function improvements in response to tirzepatide. This can help guide clinical decisions and prescribing practices, particularly when improvement in physical function is a key treatment objective.

## 2 Methods

Table S1 lists key features of the SURMOUNT‐1, ‐3, and ‐4 study design and of the current post hoc analysis.

### 2.1 Study Design and Population

The SURMOUNT‐1, ‐3, and ‐4 phase 3, multicenter, randomized, placebo‐controlled, double‐blind trials evaluated the efficacy and safety of tirzepatide for chronic weight management [22].

Adult participants (≥ 18 years old) with obesity (body mass index [BMI] ≥ 30 kg/m^2^ or ≥ 27 kg/m^2^ and ≥ 1 ORCs) and a history of ≥ 1 self‐reported unsuccessful dietary attempt to lose body weight were included in these trials [22]. The detailed study design, eligibility criteria, and endpoints for these trials have been published previously [15, 16, 17].

All trials were conducted in accordance with the good clinical practice guidelines and the principles of the Declaration of Helsinki. Each of the participating sites received approval from an independent ethics committee or institutional review board. All participants provided written informed consent prior to trial participation.

### 2.2 Randomization and Treatments

In SURMOUNT‐1, 2539 participants were randomly assigned (1:1:1:1) to receive tirzepatide (5, 10, or 15 mg) or placebo for 72 weeks [15]. In SURMOUNT‐3, 579 participants who achieved ≥ 5.0% weight reduction after a 12‐week intensive lifestyle modification program were randomly assigned (1:1) to receive tirzepatide maximum tolerated dose (MTD: 10 or 15 mg) or placebo for 72 weeks [16]. In SURMOUNT‐4, 670 participants who attained tirzepatide MTD during the 36‐week lead‐in period were randomly assigned (1:1) to continue receiving tirzepatide MTD or switch to placebo for an additional 52 weeks to assess maintenance of weight reduction [17]. In all three SURMOUNT (1, 3, and 4) trials, tirzepatide was administered subcutaneously once weekly. All participants received study treatment as an adjunct to lifestyle counseling (500 kcal/day deficit diet and ≥ 150 min of physical activity/week) [22].

### 2.3 Study Outcomes and Assessments

Patient‐reported physical function was assessed through Short Form‐36 Version 2 Health Survey acute form (SF‐36v2) Physical Functioning domain score and the Impact of Weight on Quality of Life‐Lite‐Clinical Trials Version (IWQOL‐Lite‐CT) Physical Function composite score (Table S2) in SURMOUNT‐1, ‐3, and ‐4 trials. In this post hoc analysis, participants were categorized into quartiles (Q) based on baseline SF‐36v2 Physical Functioning scores and the IWQOL‐Lite‐CT Physical Function scores within each study. The lower the quartile, the higher the degree of physical function limitation.

The study outcomes assessed by baseline quartiles of SF‐36v2 Physical Functioning domain scores and the IWQOL‐Lite‐CT Physical Function composite scores included (i) duration of obesity and presence of ORCs at baseline; (ii) efficacy measures (change from baseline in body weight, waist circumference, and BMI); and (iii) PRO measures (change from baseline in SF‐36v2 domain scores; and IWQOL‐Lite‐CT Total score and Physical Function and Psychosocial composite scores). Additionally, the correlation between weight reduction and improvement in SF‐36v2 Physical Functioning scores was assessed by baseline quartiles of SF‐36v2 Physical Functioning scores.

### 2.4 Statistical Analyses

This post hoc analysis utilized data from Weeks 0 to 72 in SURMOUNT‐1 and SURMOUNT‐3 and from Weeks 0 to 88 in SURMOUNT‐4. SURMOUNT‐1 was the primary focus of the current analysis given its largest trial sample size. SURMOUNT‐3 and SURMOUNT‐4 evaluated the consistency of the primary findings. As tirzepatide MTD arm in SURMOUNT‐3 and SURMOUNT‐4 was predominantly 15 mg, only results from the 15 mg arm in SURMOUNT‐1 are presented for efficacy and PRO measures. For SURMOUNT‐4, only results of the tirzepatide arm are reported since participants in the comparator arm received both tirzepatide (Weeks 0–36) and placebo (Weeks 36–88).

Statistical analyses were conducted using the R Version 4.2.2 software. Efficacy and PRO measures were assessed in the efficacy analysis set (randomized population who received at least one dose of the study drug, excluding data after study drug discontinuation). All study outcomes were assessed among participants with baseline SF‐36v2 Physical Functioning score or IWQOL‐Lite‐CT Physical Function composite score.

Baseline clinical characteristics were compared across quartiles using analysis of variance (ANOVA) for continuous variables and chi‐square test for categorical variables. For efficacy and PRO measures, treatments were compared (tirzepatide versus placebo) using analysis of covariance (ANCOVA) within each quartile controlling for stratification factors and baseline value of the outcome, with the last observation carried forward for missing data imputation. Pearson's correlation (*r*) between weight reduction and improved physical function was calculated using pooled data from the tirzepatide treatment arm in SURMOUNT‐1, ‐3, and ‐4. The correlation thresholds were defined as < 0.25 for weak, 0.25–0.50 for mild, 0.50–0.75 for moderate, and > 0.75 for strong. The SURMOUNT‐1, ‐3, and ‐4 trials were not powered for the current post hoc analysis; thus, the reported *p* values should be regarded as exploratory rather than confirmatory.

## 3 Results

### 3.1 Baseline Demographics and Clinical Characteristics

Baseline demographics and clinical characteristics were generally similar across SURMOUNT‐1 (*N* = 2539), SURMOUNT‐3 (*N* = 579), and SURMOUNT‐4 (*N* = 670) trials (Table 1). The participants had a mean age of 44.9 to 47.7 years, with the majority being females (62.9% to 70.6%). The mean BMI ranged from 35.9 to 38.4 kg/m^2^, mean waist circumference from 109.4 to 115.2 cm, and the mean body weight was between 101.9 and 107.3 kg. Participants had obesity for a mean duration of 14.4 to 15.5 years (Table 1).

**TABLE 1:** Baseline demographics and clinical characteristics.

Characteristics	SURMOUNT‐1 (*N* = 2539)	SURMOUNT‐3 (*N* = 579)	SURMOUNT‐4 (*N* = 670)
Age (years), mean (SD)	44.9 (12.5)	45.6 (12.2)	47.7 (12.6)
Female, *n* (%)	1714 (67.5)	364 (62.9)	473 (70.6)
Duration of obesity (years), mean (SD)	14.4 (10.8)	15.1 (11.2)	15.5 (11.8)
Body weight (kg), mean (SD)	104.8 (22.1)	101.9 (21.4)	107.3 (22.3)
BMI (kg/m^2^), mean (SD)	38.0 (6.8)	35.9 (6.3)	38.4 (6.6)
Waist circumference (cm), mean (SD)	114.1 (15.2)	109.4 (15.0)	115.2 (14.5)
Obesity‐related complications^a^, *n* (%)			
Hypertension	819 (32.3)	199 (34.4)	236 (35.2)
Anxiety or depression	422 (16.6)	116 (20.0)	151 (22.5)
Osteoarthritis	326 (12.8)	91 (15.7)	133 (19.9)
Asthma or COPD	267 (10.5)	52 (9.0)	69 (10.3)
Obstructive sleep apnea	197 (7.8)	59 (10.2)	81 (12.1)
Patient‐reported outcomes, mean (SD)			
SF‐36 v2 domain scores^b^			
Physical Functioning	49.6 (7.8)	51.7 (6.7)	47.6 (8.2)
Role‐Physical	51.4 (7.4)	53.0 (6.4)	50.1 (7.9)
Bodily Pain	52.1 (8.8)	52.6 (7.9)	50.4 (9.0)
General Health	52.4 (8.1)	54.5 (7.5)	50.7 (8.1)
Vitality	54.7 (8.2)	56.2 (7.7)	52.3 (8.4)
Social Functioning	52.5 (7.1)	53.3 (6.2)	51.7 (7.5)
Role‐Emotional	50.7 (8.3)	51.6 (7.2)	49.5 (8.9)
Mental Health	53.6 (7.3)	54.1 (6.8)	52.6 (7.8)
IWQOL‐Lite‐CT^c^			
Total score	63.1 (21.1)	68.8 (20.8)	58.0 (22.4)
Physical Function composite score	63.4 (24.0)	71.9 (22.3)	59.1 (24.5)
Psychosocial composite score	63.2 (22.8)	68.0 (22.5)	57.5 (24.3)

*Note*: Baseline was randomization (Week 0) for SURMOUNT‐1 and ‐3 and lead‐in baseline (Week 0) for SURMOUNT‐4. *N* may vary across variables due to missing values.

Abbreviations: COPD, chronic obstructive pulmonary disorder; IWQOL‐Lite‐CT, Impact of Weight on Quality of Life‐Lite‐Clinical Trials Version; SF‐36v2, Short Form‐36 Version 2 Health Survey acute form.

^a^ Baseline medical conditions were assessed through a review of participants' medical history.

^b^ SF‐36v2 measures health‐related quality of life and general health status. The SF‐36v2 scores are norm‐based scores, with the 2009 US general population mean at 50 and an SD of 10. Higher scores indicate better health status.

^c^ IWQOL‐Lite‐CT measures weight‐specific health‐related quality of life. All items are rated on a 5‐point frequency scale (“never” to “always”) or a 5‐point truth scale (“not at all true” to “completely true”). Scores range from 0 to 100, with higher scores indicating better functioning.

Table 2 presents the quartiles of SF‐36v2 Physical Functioning scores and IWQOL‐Lite‐CT Physical Function composite scores at baseline. The baseline scores were comparable between the tirzepatide and placebo groups across quartiles within each trial. The distribution of the baseline SF‐36v2 Physical Functioning scores varied across the trials, with SURMOUNT‐3 demonstrating the highest degree of ceiling effect (i.e., achieving highest score): SURMOUNT‐4 (Q1, median, Q3) = 42.2, 49.9, 53.7; SURMOUNT‐1 = 46.0, 51.8, 55.7; and SURMOUNT‐3 = 49.9, 53.7, 57.6. Similarly, SURMOUNT‐3 had the highest degree of ceiling effect for the baseline IWQOL‐Lite‐CT Physical Function composite scores: SURMOUNT‐4 (Q1, median, Q3) = 40.0, 60.0, 80.0; SURMOUNT‐1 = 45.0, 65.0, 85.0; and SURMOUNT‐3 = 60.0, 75.0, 90.0.

**TABLE 2:** Baseline quartiles of SF‐36v2 Physical Functioning domain score and IWQOL‐Lite‐CT Physical Function composite score (randomized population).

	SURMOUNT‐1			SURMOUNT‐3			SURMOUNT‐4		
	Placebo (*N* = 643)	Tirzepatide pooled (*N* = 1896)	Total (*N* = 2539)	Placebo (*N* = 292)	Tirzepatide MTD (*N* = 287)	Total (*N* = 579)	Placebo (*N* = 335)	Tirzepatide MTD (*N* = 335)	Total (*N* = 670)
SF‐36v2 Physical Functioning domain score (norm‐based)									
*n*	643	1892	2535	290	285	575	335	335	670
Min	21.0	19.0	19.0	19.0	21.0	19.0	21.0	21.0	21.0
Q1	46.0	46.0	46.0	49.9	48.0	49.9	42.2	42.2	42.2
Median	51.8	51.8	51.8	53.7	53.7	53.7	49.9	49.9	49.9
Q3	55.7	55.7	55.7	57.6	57.6	57.6	53.7	53.7	53.7
Max	57.6	57.6	57.6	57.6	57.6	57.6	57.6	57.6	57.6
IWQOL‐Lite‐CT Physical Function composite score									
*n*	639	1891	2530	291	287	578	335	335	670
Min	0.0	0.0	0.0	0.0	0.0	0.0	0.0	0.0	0.0
Q1	45.0	45.0	45.0	60.0	60.0	60.0	40.0	45.0	40.0
Median	65.0	65.0	65.0	75.0	80.0	75.0	65.0	60.0	60.0
Q3	85.0	85.0	85.0	90.0	90.0	90.0	80.0	80.0	80.0
Max	100.0	100.0	100.0	100.0	100.0	100.0	100.0	100.0	100.0

Abbreviations: IWQOL‐Lite‐CT, Impact of Weight on Quality of Life‐Lite‐Clinical Trials Version; max, maximum; min, minimum; MTD, maximum tolerated dose; Q1, first quartile; Q3, third quartile; SF‐36v2, Short Form‐36 Version 2 Health Survey acute form.

### 3.2 Clinical Characteristics Associated With Lower Physical Function

**FIGURE 1:**
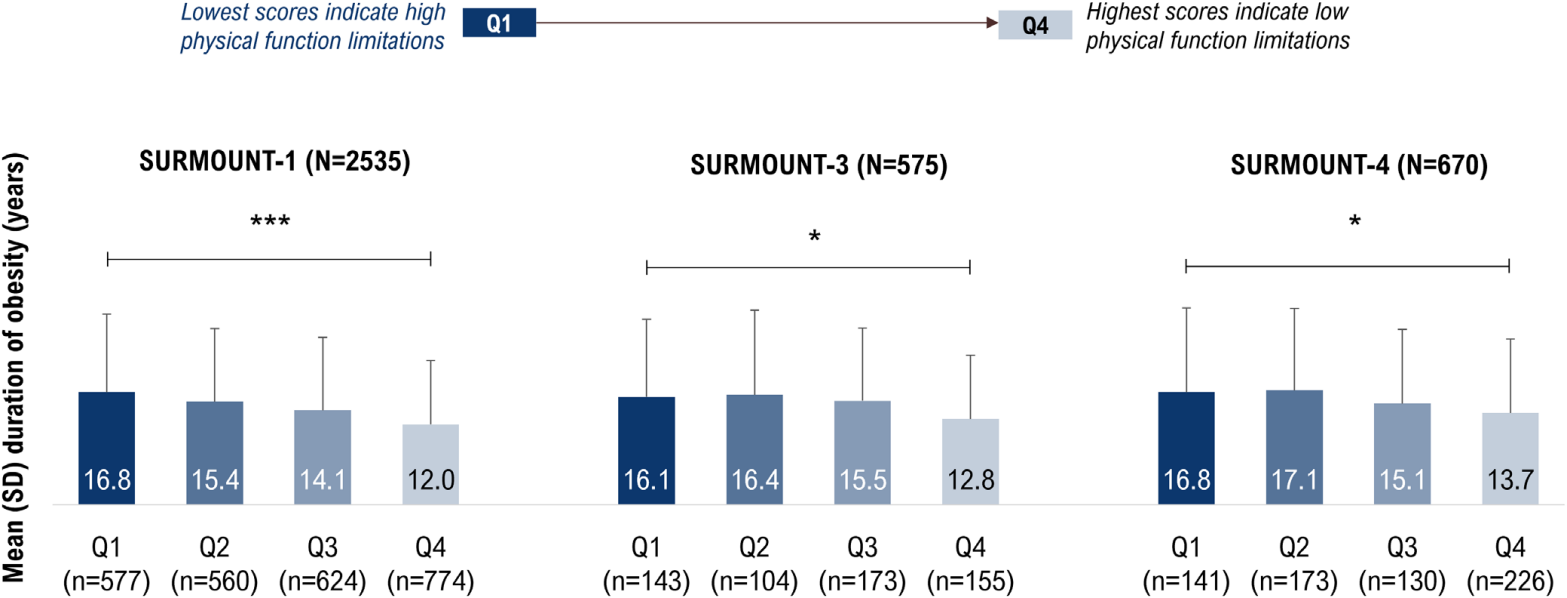
Duration of obesity by baseline quartiles of SF‐36v2 Physical Functioning domain score. *p < 0.05; ***p < 0.001 across baseline quartiles based on ANOVA model. Baseline is randomization (Week 0) for SURMOUNT‐1 and SURMOUNT‐3 and lead‐in baseline (Week 0) for SURMOUNT‐4. Q1, first quartile; Q2, second quartile; Q3, third quartile; Q4, fourth quartile; SF‐36v2, Short Form‐36 Version 2 Health Survey acute form. [Color figure can be viewed at wileyonlinelibrary.com]

Participants with lower baseline SF‐36v2 Physical Functioning scores had a longer duration of obesity (SURMOUNT‐1: Q1 to Q4 mean = 16.8 to 12.0 years, *p* < 0.001 for comparison across the quartiles; SURMOUNT‐3: 16.1 to 12.8 years, *p* < 0.05; SURMOUNT‐4: 16.8 to 13.7 years, *p* < 0.05) (Figure 1).

Similarly, participants who had lower IWQOL‐Lite‐CT Physi cal Function composite scores at baseline had a longer duration of obesity in SURMOUNT‐1 (Q1 to Q4 mean = 17.2 to 12.2 years, *p* < 0.001), SURMOUNT‐3 (17.3 to 13.9 years, *p* = 0.055), and SURMOUNT‐4 (17.1 to 13.5 years, *p* < 0.05) (Figure S1).

**FIGURE 2:**
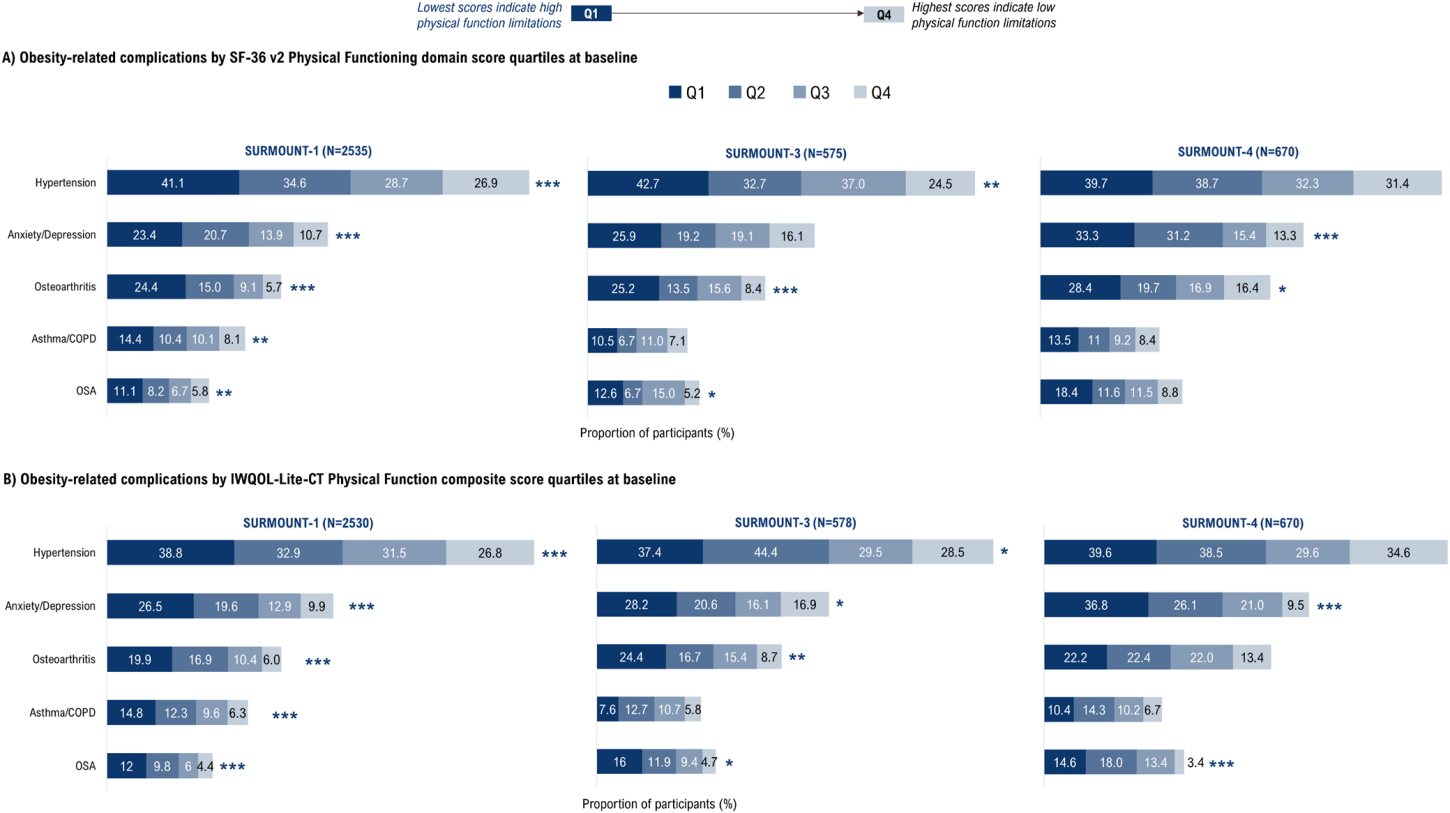
Obesity‐related complications. *p < 0.05; **p < 0.01; ***p < 0.001 across baseline quartiles based on chi‐square test. Baseline is randomization (Week 0) for SURMOUNT‐1 and SURMOUNT‐3 and lead‐in baseline (Week 0) for SURMOUNT‐4. COPD, chronic obstructive pulmonary disorder; IWQOL‐Lite‐CT, Impact of Weight on Quality of Life‐Lite‐Clinical Trials Version; OSA, obstructive sleep apnea; Q1, first quartile; Q2, second quartile; Q3, third quartile; Q4, fourth quartile; SF‐36v2, Short Form‐36 Version 2 Health Survey acute form. [Color figure can be viewed at wileyonlinelibrary.com]

Lower baseline SF‐36v2 Physical Functioning scores and IWQOL‐Lite‐CT Physical Function composite scores were associated with a higher prevalence of ORCs including hypertension, anxiety/depression, osteoarthritis, asthma/chronic obstructive pulmonary disease (COPD), and OSA in SURMOUNT trials (Figure 2).

### 3.3 Efficacy Measures by Baseline Quartiles of Physical Function

**FIGURE 3:**
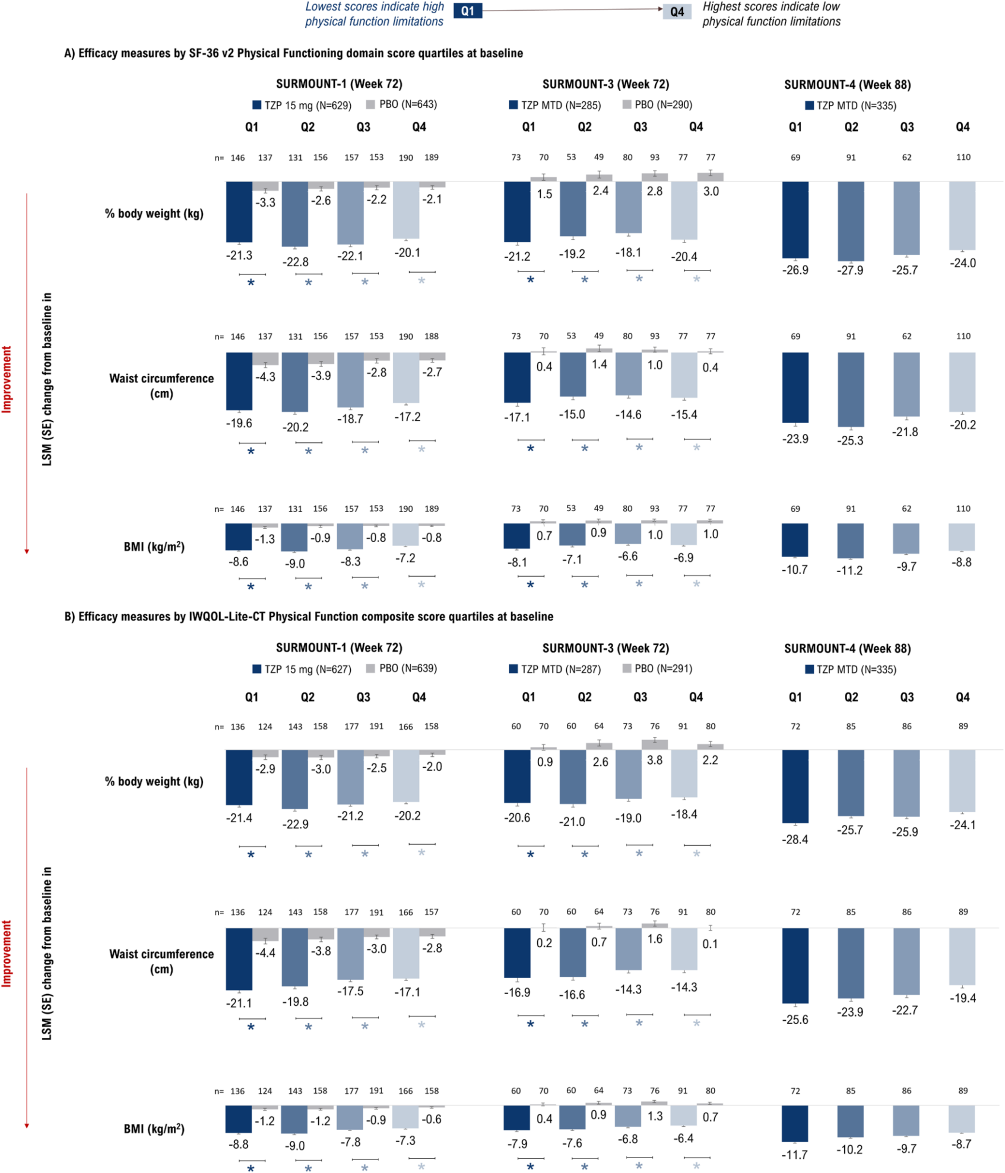
Change from baseline in efficacy measures at end of study. *p < 0.05 versus placebo. Data are presented as LSM (SE) change from baseline (randomization [Week 0] for SURMOUNT‐1 and ‐3 and lead‐in baseline [Week 0] for SURMOUNT‐4) at Week 72 (SURMOUNT‐1 and ‐3) and Week 88 (SURMOUNT‐4) using ANCOVA with LOCF. In SURMOUNT‐4, the comparator arm received both tirzepatide (Weeks 0–36) and placebo (Weeks 36–88). Thus its results are not presented. IWQOL‐Lite‐CT, Impact of Weight on Quality of Life‐Lite‐Clinical Trials Version; LOCF, last observation carried forward; LSM, least‐squares mean; MTD, maximum tolerated dose; PBO, placebo; Q1, first quartile; Q2, second quartile; Q3, third quartile; Q4, fourth quartile; SF‐36v2, Short Form‐36 Version 2 Health Survey acute form; TZP, tirzepatide. [Color figure can be viewed at wileyonlinelibrary.com]

Tirzepatide treatment was associated with greater reductions in body weight, BMI, and waist circumference compared to placebo across all quartiles of SF‐36v2 Physical Functioning (Figure 3A) and IWQOL‐Lite‐CT Physical Function (Figure 3B) composite scores (*p* < 0.05).

The least‐squares mean (LSM) percent changes in body weight (kg) with tirzepatide vs. placebo were consistent across baseline quartiles of SF‐36v2 Physical Functioning scores in SURMOUNT‐1 (Week 72: −20.1% to −22.8% vs. −2.1% to −3.3%), SURMOUNT‐3 (Week 72: −18.1% to −21.2% vs. 1.5% to 3.0%), and SURMOUNT‐4 (Week 88 [tirzepatide]: −24.0% to −27.9%) (Figure 3A). Similarly, the LSM changes in waist circumference were comparable across quartiles in SURMOUNT‐1 (tirzepatide vs. placebo: −17.2 to −20.2 cm vs. −2.7 to −4.3 cm), SURMOUNT‐3 (−14.6 to −17.1 cm vs. 0.4 to 1.4 cm), and SURMOUNT‐4 (−20.2 to −25.3 cm) (Figure 3A). The LSM changes in BMI also followed a similar pattern: SURMOUNT‐1 (−7.2 to −9.0 kg/m^2^ vs. −0.8 to −1.3 kg/m^2^), SURMOUNT‐3 (−6.6 to −8.1 kg/m^2^ vs. 0.7 to 1.0 kg/m^2^), and SURMOUNT‐4 (−8.8 to −11.2 kg/m^2^) (Figure 3A).

At Week 72, the LSM percent changes in body weight (kg) with tirzepatide vs. placebo were similar across baseline quartiles of IWQOL‐Lite‐CT Physical Function composite scores in SURMOUNT‐1 (−20.2% to −22.9% vs. −2.0% to −3.0%), SURMOUNT‐3 (−18.4% to −21.0% vs. 0.9% to 3.8%), and SURMOUNT‐4 (tirzepatide at Week 88: −24.1% to −28.4%) (Figure 3B). Similarly, the LSM changes in waist circumference were consistent across quartiles in SURMOUNT‐1 (tirzepatide vs. placebo: −17.1 to −21.1 cm vs. −2.8 to −4.4 cm), SURMOUNT‐3 (−14.3 to −16.9 cm vs. 0.1 to 1.6 cm), and SURMOUNT‐4 (tirzepatide: −19.4 to −25.6 cm). The LSM changes in BMI followed a similar pattern, with comparable reductions across quartiles with tirzepatide vs. placebo in SURMOUNT‐1 (−7.3 to −9.0 kg/m^2^ vs. −0.6 to −1.2 kg/m^2^), SURMOUNT‐3 (−6.4 to −7.9 kg/m^2^ vs. 0.4 to 1.3 kg/m^2^), and SURMOUNT‐4 (tirzepatide at Week 88: −8.7 to −11.7 kg/m^2^) (Figure 3B).

### 3.4 PRO Measures by Baseline Quartiles of Physical Function

**FIGURE 4:**
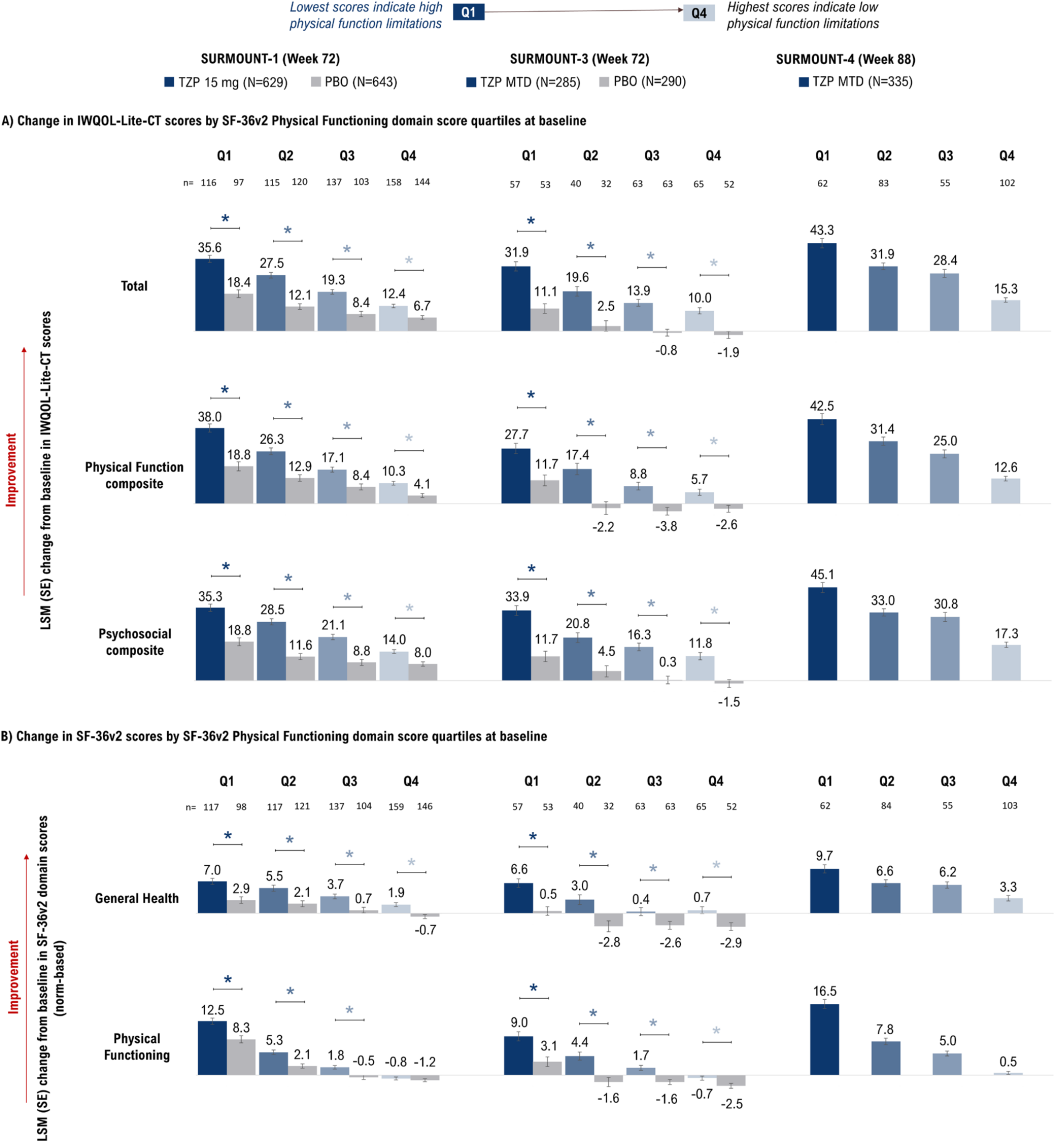
Change from baseline in patient‐reported outcomes at end of study by baseline quartiles of SF‐36v2 Physical Functioning domain score. *p < 0.05 versus placebo. Data are presented as LSM (SE) change from baseline (randomization [Week 0] for SURMOUNT‐1 and ‐3 and lead‐in baseline [Week 0] for SURMOUNT‐4) at Week 72 (SURMOUNT‐1 and ‐3) and Week 88 (SURMOUNT‐4) using ANCOVA with LOCF. In SURMOUNT‐4, the comparator arm received both tirzepatide (Weeks 0–36) and placebo (Weeks 36–88). Thus its results are not presented. IWQOL‐Lite‐CT, Impact of Weight on Quality of Life‐Lite‐Clinical Trials Version; LOCF, last observation carried forward; LSM, least‐squares mean; MTD, maximum tolerated dose; PBO, placebo; PRO, patient‐reported outcome; Q1, first quartile; Q2, second quartile; Q3, third quartile; Q4, fourth quartile; SF‐36v2, Short Form‐36 Version 2 Health Survey acute form; TZP, tirzepatide. [Color figure can be viewed at wileyonlinelibrary.com]

**FIGURE 5:**
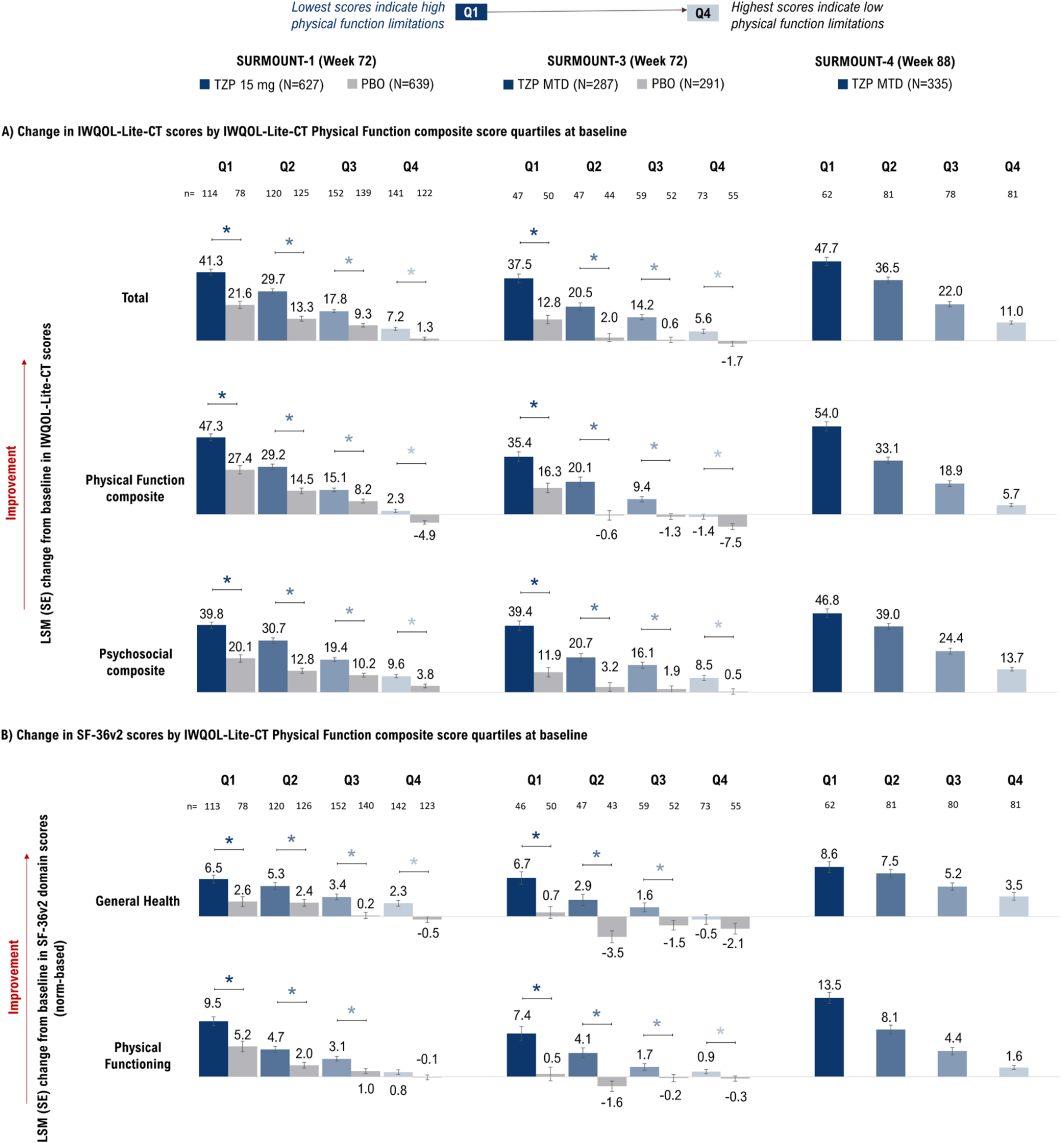
Change from baseline in patient‐reported outcomes at the end of the study by baseline quartiles of IWQOL‐Lite‐CT Physical Function composite score. *p < 0.05 versus placebo. Data are presented as LSM (SE) change from baseline (randomization [Week 0] for SURMOUNT‐1 and ‐3 and lead‐in baseline [Week 0] for SURMOUNT‐4) at Week 72 (SURMOUNT‐1 and ‐3) and Week 88 (SURMOUNT‐4) using ANCOVA with LOCF. In SURMOUNT‐4, the comparator arm received both tirzepatide (Weeks 0–36) and placebo (Weeks 36–88). Thus its results are not presented. IWQOL‐Lite‐CT, Impact of Weight on Quality of Life‐Lite‐Clinical Trials Version; LOCF, last observation carried forward; LSM, least‐squares mean; MTD, maximum tolerated dose; PBO, placebo; PRO, patient‐reported outcome; Q1, first quartile; Q2, second quartile; Q3, third quartile; Q4, fourth quartile; SF‐36v2, Short Form‐36 Version 2 Health Survey acute form; TZP, tirzepatide. [Color figure can be viewed at wileyonlinelibrary.com]

Despite similar changes in efficacy measures across baseline quartiles of physical function, improvements in physical function were more pronounced with tirzepatide among participants with lower baseline physical function. Particularly, participants with lower baseline SF‐36v2 Physical Functioning scores (Q1) achieved greater LSM improvements in IWQOL‐Lite‐CT Physical Function composite scores in SURMOUNT‐1 (Week 72 [tirzepatide vs. placebo]: Q1 = 38.0 vs. 18.8; Q4 = 10.3 vs. 4.1; *p* < 0.05 for tirzepatide vs. placebo in all quartiles), SURMOUNT‐3 (Week 72 [tirzepatide vs. placebo]: Q1 = 27.7 vs. 11.7; Q4 = 5.7 vs. −2.6; all *p* < 0.05), and SURMOUNT‐4 (tirzepatide; Week 88: Q1 = 42.5; Q4 = 12.6) (Figure 4A). Similarly, participants with lower baseline SF‐36v2 Physical Functioning scores achieved greater LSM improvements in these scores after tirzepatide treatment in SURMOUNT‐1 (Q1 = 12.5 vs. 8.3; Q4 = −0.8 vs. −1.2; all *p* < 0.05 except Q4), SURMOUNT‐3 (Q1 = 9.0 vs. 3.1; Q4 = −0.7 vs. −2.5; all *p* < 0.05), and SURMOUNT‐4 (tirzepatide: Q1 = 16.5; Q4 = 0.5) (Figure 4B). These findings for improvement in physical function were consistent for baseline quartiles of IWQOL‐Lite‐CT Physical Function composite scores (Figure 5).

Participants with lower baseline SF‐36v2 Physical Functioning scores achieved greater LSM improvements in IWQOL‐Lite‐CT Psychosocial composite scores in SURMOUNT‐1 (Week 72 [tirzepatide vs. placebo]: Q1 = 35.3 vs. 18.8; Q4 = 14.0 vs. 8.0; all *p* < 0.05), SURMOUNT‐3 (Week 72 [tirzepatide vs. placebo]: Q1 = 33.9 vs. 11.7; Q4 = 11.8 vs. −1.5; all *p* < 0.05), and SURMOUNT‐4 (tirzepatide; Week 88: Q1 = 45.1; Q4 = 17.3) (Figure 4A).

Participants with lower baseline SF‐36v2 Physical Functioning scores achieved greater LSM improvements in IWQOL‐Lite‐CT Total score after tirzepatide treatment in SURMOUNT‐1 (Q1 = 35.6 vs. 18.4; Q4 = 12.4 vs. 6.7; all *p* < 0.05), SURMOUNT‐3 (Q1 = 31.9 vs. 11.1; Q4 = 10.0 vs. −1.9; all *p* < 0.05), and SURMOUNT‐4 (tirzepatide: Q1 = 43.3; Q4 = 15.3) (Figure 4A). Improvements in SF‐36v2 General Health were also more pronounced in participants with lower baseline SF‐36v2 Physical Functioning scores in SURMOUNT‐1 (Q1 = 7.0 vs. 2.9; Q4 = 1.9 vs. −0.7; all *p* < 0.05), SURMOUNT‐3 (Q1 = 6.6 vs. 0.5; Q4 = 0.7 vs. −2.9; all *p* < 0.05), and SURMOUNT‐4 (tirzepatide: Q1 = 9.7; Q4 = 3.3) (Figure 4B). These improvements in IWQOL‐Lite‐CT Total and Psychosocial composite scores, and SF‐36v2 General Health were consistent for baseline quartiles of IWQOL‐Lite‐CT Physical Function composite scores (Figure 5).

Participants with lower baseline physical function scores showed greater improvements in other SF‐36v2 domain scores (Role‐Physical, Bodily Pain, Vitality, Social Functioning, Role‐Emotional, and Mental Health) after tirzepatide treatment (Table S3).

### 3.5 Correlation Between Weight Reduction and Improved Physical Function

Based on quartiles of baseline SF‐36v2 Physical Functioning scores, a weak to mild correlation was observed between weight reduction and improved SF‐36v2 Physical Functioning scores among tirzepatide‐treated participants pooled from SURMOUNT‐1, ‐3, and ‐4. The strength of correlation decreased from Q1 to Q4 (Q1: *r* = −0.26; Q2: *r* = −0.20; Q3: *r* = −0.19; Q4: *r* = −0.04).

## 4 Discussion

This post hoc analysis found that tirzepatide treatment was associated with substantial weight reduction across all levels of baseline physical function, with the greatest improvements in physical function observed in individuals with the most severe physical function limitations. As physical function is linked to quality of life and work productivity, improvements in physical function with tirzepatide treatment may improve quality of life and work productivity, particularly in patients with greater baseline physical function impairments. Furthermore, this analysis identified characteristics of obesity beyond weight (duration of obesity, ORCs) that are associated with impaired physical function. Specifically, participants with lower baseline physical function had a longer duration of obesity and a higher prevalence of ORCs (hypertension, anxiety/depression, osteoarthritis, asthma/COPD, and OSA).

Obesity is associated with accelerated age‐related functional decline. Adults with obesity (BMI ≥ 30.0 kg m^2^) experience nearly twice the physical function decline compared to those with normal weight (BMI < 25 kg/m^2^), and are almost four times more likely to develop mobility limitations and disabilities [23]. Multimorbidity is also linked with a decline in physical function, decreased HRQoL, and higher mortality rates [24, 25]. The current analysis found that participants with lower baseline physical function had a higher prevalence of ORCs. These findings highlight the importance of early weight management and regular monitoring of physical function in people with obesity [26, 27].

Participants in SURMOUNT trials reported a mean body weight reduction of ≥ 15% with tirzepatide, regardless of their baseline physical function. Notably, despite comparable levels of weight reduction across all quartiles of baseline physical function, participants with lower baseline physical function experienced greater improvements in physical function. These findings suggest that (1) limitations in physical function do not inhibit tirzepatide‐led weight reduction, indicating weight reduction is independent of physical function limitations, and (2) the perceived improvements in physical function associated with tirzepatide are related to the extent of the initial deficit. These observations warrant further investigation to characterize the magnitude and underlying mechanisms of these apparent benefits.

In SURMOUNT‐1, meaningful within‐participant changes of ≥ 5.76 for the SF‐36v2 Physical Functioning domain score and ≥ 25 for the IWQOL‐Lite‐CT Physical Function composites score were empirically determined using anchor‐based and distribution‐based methods [18]. In the current analysis, tirzepatide‐treated participants in the lowest baseline physical function quartile achieved mean improvements that exceeded these clinically meaningful thresholds. These findings highlight the importance of providing treatment for individuals with lower baseline physical function, as they show meaningful improvements in both weight reduction and physical function.

Based on quartiles of baseline physical function, a weak to mild correlation was observed between weight reduction and improved physical function, consistent with those observed in SURMOUNT‐1 [18]. These findings suggest that tirzepatide may improve physical function through both weight loss‐independent and weight loss‐dependent mechanisms. The effect of tirzepatide on physical function is supported by existing studies. For example, a post hoc analysis of SURMOUNT‐1 demonstrated that greater fat mass loss, measured by dual‐energy x‐ray absorptiometry, was associated with greater improvements in physical function regardless of age [28, 29].

In the ARMMS‐T2D study in people with obesity and T2D, the improvement in SF‐36 PCS scores was significantly greater with metabolic/bariatric surgery versus medical/lifestyle intervention (*p* < 0.001) over 12 years. Additionally, BMI reduction was greater after metabolic/bariatric surgery versus medical/lifestyle intervention (*p* < 0.001) and correlated with improved PCS [30]. Tirzepatide primarily reduces fat mass, improving physical function and metabolic health [15, 31]. These effects are more similar to bariatric surgery than diet, as bariatric surgery essentially acts as a physiological intervention rather than causing nutrient malabsorption. Tirzepatide suppresses appetite, reduces food intake, cravings, and preferences, enhances satiety, and potentially increases energy expenditure [32, 33, 34]. These effects differentiate tirzepatide from dietary interventions, which often fail to overcome food noise, leading to weight regain and diminished physical function [31, 35]. These multifaceted mechanisms of tirzepatide may contribute to improvements in physical function and various other aspects of HRQoL, independent of weight loss.

### 4.1 Strengths

This is one of the first papers to look at physical functioning from a physiologically induced versus weight loss‐dependent mechanism. Although the degree of ceiling effect varied across the trials, the study findings and trends remained consistent, allowing a reasonable degree of generalization. Similarly, the use of the multiple SURMOUNT phase 3 trials enabled the cross‐study evaluation on whether the observed between‐quartile differences in efficacy measures were artifacts or systemic. We utilized both generic (SF‐36v2) and disease‐specific (IWQOL‐Lite‐CT) PROs to categorize participants based on their baseline physical function, and consistent results were observed across these PROs. This study found that the degree of physical dysfunction at baseline is a determinant of the extent of improvement. The results suggest that individuals with worse perceived physical functioning at baseline have greater potential for recovery, and the data confirm that such improvement is achievable, regardless of the initial degree of impairment. However, this is not always seen in other severe baseline conditions. For instance, in diabetes management, individuals with high HbA1c levels may achieve smaller relative improvements (the change of mean HbA1c level divided by mean HbA1c level before therapy) due to more complex physiological or behavioral barriers than those with moderately elevated levels [36]. Similarly, people with severe baseline physical impairments, like advanced joint degeneration, may experience less improvement in mobility after physical therapy compared to those with milder impairments due to their more limited recovery potential [37]. These findings are clinically relevant, as they underscore the importance of the observed inverse relationship and highlight the need for further investigation into its underlying mechanisms and predictors of improvement in physical functioning. A better understanding of these determinants can support more effective treatment decision‐making, particularly when enhancing physical function is a key objective.

### 4.2 Limitations

As this was a post hoc analysis, the findings are hypothesis generating rather than confirming. The study relied on self‐reported physical functioning instead of objective measures of physical functioning. Measuring both actual physical functioning and reported physical functioning scores in future studies can provide a better understanding of the effect of obesity medications on physical function decline, a symptom commonly associated with obesity. Furthermore, integrating these measurements with the assessment of changes in muscle mass and muscle function can help elucidate the relationship between the decline in actual physical function and the decline in perceived physical function. Understanding this relationship is important because various factors, such as improved quality of life and mental health, can contribute to perceived improvements in physical functioning.

## 5 Conclusion

Tirzepatide treatment was associated with substantial weight reduction in adults with obesity, regardless of baseline physical function limitations, indicating a degree of independence between physical function status and weight reduction. Moreover, individuals with more severe physical function impairments at baseline experienced greater improvements in physical function, highlighting that those with the greatest limitations may derive the most benefit from treatment. Therefore, prioritizing therapies that enhance physical function may be particularly important for this population. Our findings also suggest that improvements in physical function may be mediated not only by weight loss but also by additional physiological effects of tirzepatide. Further studies are warranted to elucidate these underlying mechanisms.

## Author Contributions

**Xuan Li:** conception and design of the work, interpretation of data for the work, and critical review of the work for important intellectual content. **Dachuang Cao:** design of the work, acquisition of data for the work, analysis and interpretation of data for the work, and critical review of the work for important intellectual content. **Helene Sapin:** interpretation of data for the work and critical review of the work for important intellectual content. **Fangyu Wang:** analysis and interpretation of data for the work, and critical review of the work for important intellectual content. **Theresa Hunter Gibble:** design of the work, interpretation of data for the work, and critical review of the work for important intellectual content. **Nedina Kalezic Raibulet:** design of the work, analysis and interpretation of data for the work, and critical review of the work for important intellectual content. **Max Denning:** conception of the work, interpretation of data for the work, and critical review of the work for important intellectual content. **Lee M. Kaplan:** analysis and interpretation of data for the work, drafting of the work, and critical review of the work for important intellectual content. All authors take responsibility for the integrity of the work as a whole and have given their approval for this version to be published.

## Conflicts of Interest

Xuan Li, Dachuang Cao, Helene Sapin, Fangyu Wang, Theresa Hunter Gibble, Nedina Kalezic Raibulet, and Max Denning are employees and stockholders of Eli Lilly and Company. Lee M. Kaplan is the consultant for Altimmune, Amgen, AstraZeneca, Bain Capital, Boehringer Ingelheim, Cytoki Pharma, Eli Lilly and Company, Gelesis, Gilead Sciences, Glyscend, Intellihealth, Johnson and Johnson, Kallyope, Novo Nordisk, Optum Health, Perspectum, Pfizer, Sidekick Health, Skye Bioscience, twenty30 Health, Xeno Biosciences, and Zealand Pharma.

## Supporting information


**Table S1:** Overview of SURMOUNT trials and current post hoc analysis.
**Table S2:** Detailed description of patient‐reported outcome measures.
**Table S3:** SF‐36v2 domain scores by baseline quartiles of SF‐36 v2 Physical Functioning domain score and IWQOL‐Lite‐CT Physical Function composite score.
**Figure S1:** Duration of obesity by baseline quartiles of IWQOL‐Lite‐CT Physical Function composite score.

## Data Availability

Lilly provides access to all individual participant data collected during the trial, after anonymization, with the exception of pharmacokinetic or genetic data. Data are available to request 6 months after the indication studied has been approved in the US and EU and after primary publication acceptance, whichever is later. No expiration date for data requests is currently set once data are made available. Access is provided after a proposal has been approved by an independent review committee identified for this purpose and after receipt of a signed data sharing agreement. Data and documents, including the study protocol, statistical analysis plan, clinical study report, and blank or annotated case report forms, will be provided in a secure data sharing environment. For details on submitting a request, see the instructions provided at www.vivli.org.
