# Supplementary material for: People With Lowest Physical Functioning Scores Showed Greatest Improvement After Tirzepatide Treatment
Source: Obesity (Silver Spring). 2025 Nov 4;34(1):114–26. doi: 10.1002/oby.70067 (PMC12724063; doi:10.1002/oby.70067)
Supplement: Supplementary file 1 — Table S1: Overview of SURMOUNT trials and current post hoc analysis. Table S2: Detailed description of patient‐reported outcome measures. Table S3: SF‐36v2 domain scores by baseline quartiles of SF‐36 v2 Physical Functioning domain score and IWQOL‐Lite‐CT Physical Function composite score. Figure S1: Duration of obesity by baseline quartiles of IWQOL‐Lite‐CT Physical Function composite score. [file OBY-34-114-s001.docx]

# **People with Lowest Physical Functioning Scores Showed Greatest Improvement after Tirzepatide Treatment**

**Running title:** Baseline physical function & outcomes in SURMOUNT

**Author list:** Xuan Li^1,^*, Dachuang Cao^1^, Helene Sapin^1^, Fangyu Wang^1^, Theresa Hunter Gibble^1^, Nedina Kalezic Raibulet^1^, Max Denning^1^, Lee M. Kaplan^2^

**Affiliations**

^1^Eli Lilly and Company, Indianapolis, IN, USA

**^2^**Geisel School of Medicine at Dartmouth and Dartmouth Health, New Hampshire, USA

***Corresponding author**

Xuan Li

Eli Lilly and Company,

893 S. Delaware Street

Indianapolis, IN, 46285, USA

e-mail: [xuan_li@lilly.com](mailto:xuan_li@lilly.com)

**Table S1. Overview of SURMOUNT trials and current post hoc analysis.**

|  | **SURMOUNT-1**  **N=2,539** | **SURMOUNT-3**  **N=579** | **SURMOUNT-4**  **N=670** |
| --- | --- | --- | --- |
| **Study overview** | | | |
| Key inclusion criteria | - Adult participants (≥18 years old) with obesity (BMI ≥30 kg/m^2^) or overweight (BMI ≥27 kg/m^2^) and ≥1 ORCs (e.g., hypertension, dyslipidemia, obstructive sleep apnea, or cardiovascular disease) - History of ≥1 self-reported unsuccessful dietary attempt to lose body weight | | |
| Key exclusion criteria | - Diabetes - Self-reported change in body weight of >5 kg or use of obesity management medication within 3 months before screening - Prior surgical treatment for obesity | | |
| Randomization and treatments | 1:1:1:1  Tirzepatide 5, 10, and 15 mg, and placebo  administered subcutaneously once weekly | 1:1  Tirzepatide MTD (10 or 15 mg) and placebo administered subcutaneously once weekly | |
|  | All participants received study treatment as an adjunct to lifestyle counseling (500 kcal/day deficit diet and at least 150 minutes of physical activity/week) | | |
| Treatment duration | 72 weeks | 72 weeks | 88 weeks (36-week open-label tirzepatide lead-in period + 52-week double-blind treatment period) |
| **Aspects of current post hoc analysis** | | | |
| Study outcomes | Assessed by baseline quartiles of SF-36v2 Physical Functioning and the IWQOL-Lite-CT Physical Function scores within each trial (lower quartile indicate higher degree of physical function limitations)   - Duration of obesity - Presence of ORCs - Efficacy measures (BMI, weight, waist circumference) - PRO measures (SF-36v2 domain scores, and IWQOL-Lite-CT Total score and Physical Function and Psychosocial composite scores)   Additionally, correlation between weight reduction and improvement in SF-36v2 Physical Functioning scores was assessed by baseline quartiles of SF-36v2 Physical Functioning scores | | |
| Rationale for analysis | Primary focus of post hoc analysis, given its large sample size | To evaluate the consistency of the primary findings | |
| Analysis population | Tirzepatide 15 mg, placebo | Tirzepatide MTD (10 or 15 mg), placebo | Tirzepatide MTD (10 or 15 mg) |
| Rationale for the treatment arms included | Consistency across trials: The tirzepatide MTD arm in SURMOUNT-3 and -4 was predominantly 15 mg. |  | For the Weeks 0 – 88 analysis, only results of the tirzepatide arm are reported since participants in the comparator arm received both tirzepatide (Weeks 0–36) and placebo (Weeks 36–88). |
| Statistical analyses | - Comparison of baseline clinical characteristics across quartiles of physical function: ANOVA (continuous variables), Chi-square test (categorical variables) - Treatment comparisons (tirzepatide versus placebo) for efficacy and PRO measures: ANCOVA with the last observation carried forward for missing data imputation - Pearson's correlation (r) between weight reduction and improved physical function was calculated using pooled data from the tirzepatide treatment arm in SURMOUNT-1, -3, and -4. | | |

Abbreviations: ANCOVA, analysis of covariance; ANOVA, analysis of variance; BMI, body mass index; IWQOL-Lite-CT, Impact of Weight on Quality of Life-Lite-Clinical Trials Version; MTD, maximum tolerated dose; ORC, obesity-related complication; PRO, patient-reported outcome; SF-36v2, Short Form-36 Version 2 Health Survey acute form

**Table S2: Detailed description of patient-reported outcome measures.**

| *Short Form-36 Version 2 Health Survey (SF-36v2) Acute Form, 1-Week Recall Version* | The SF-36v2 is a 36-item questionnaire that measures general health-related quality of life and health status. It consists of 8 domain scores: Physical Functioning; Role-Physical; Bodily Pain; General Health; Vitality; Social Functioning; Role-Emotional; and Mental Health. Each domain is scored individually, and these scores are combined into two component summary scores: Physical Component Summary (PCS) and Mental Component Summary (MCS).  The Physical Functioning domain assesses limitations due to health “now,” while the remaining domains assess functioning “in the last week.” Items are scored on Likert scales of varying lengths (3-, 5-, or 6-point). The SF-36v2 scores are norm-based scores, with the 2009 US general population mean at 50 and an SD of 10. Higher scores indicate better health status.^1^ |
| --- | --- |
| *Impact of Weight on Quality of Life-Lite for Clinical Trials Version (IWQOL-Lite-CT)* | The IWQOL-Lite-CT is a 20-item PRO measure used to assess weight-related quality of life. It assesses two primary domains: physical composite (7 items) and psychosocial composite (13 items). A 5-item subset of the Physical composite, the Physical Function composite, is also assessed. Items in the Physical Function composite describe physical impacts related to general and specific physical activities. All items are rated on either a 5-point frequency (“never” to “always”) or a 5-point truth (“not at all true” to “completely true”) scale. The overall score range is from 0 to 100 with higher scores indicating better functioning.^2,3^ |

Abbreviations: FDA, Food and Drug Administration; HRQoL, health-related quality of life; PRO, patient-reported outcome; SD, standard deviation.

**Table S3. SF-36v2 domain scores by baseline quartiles of SF-36 v2 Physical Functioning domain Score and IWQOL-Lite-CT Physical Function composite score.**

|  | SURMOUNT-1  TZP 15mg: N=629; PBO: N=643 | | | | | | | | SURMOUNT-3  TZP MTD: N=285; PBO: N=290 | | | | | | | | SURMOUNT-4 TZP MTD: N=335 | | | |
| --- | --- | --- | --- | --- | --- | --- | --- | --- | --- | --- | --- | --- | --- | --- | --- | --- | --- | --- | --- | --- |
|  | Q1 | | Q2 | | Q3 | | Q4 | | Q1 | | Q2 | | Q3 | | Q4 | | Q1 | Q2 | Q3 | Q4 |
|  | TZP  15 mg | PBO | TZP  15 mg | PBO | TZP  15 mg | PBO | TZP  15 mg | PBO | TZP MTD | PBO | TZP MTD | PBO | TZP MTD | PBO | TZP MTD | PBO | TZP MTD | | | |
| **Results by SF-36v2 Physical Functioning domain Score quartiles at baseline** | | | | | | | | | | | | | | | | | | | | |
| **LSM (SE) change from baseline in SF-36 v2 domain scores** | | | | | | | |  |  |  |  |  |  |  |  |  |  |  |  |  |
| Role-Physical | 8.6* (0.69) | 5.0 (0.75) | 3.6* (0.54) | 1.8 (0.53) | 0.6 (0.47) | 0.2 (0.54) | -0.1 (0.32) | -0.1 (0.33) | 6.5* (0.97) | 2.5 (1.01) | 0.9 (0.89) | -1.5 (1.00) | -0.1 (0.75) | -1.3 (0.75) | 0.2* (0.58) | -3.1 (0.65) | 11.2 (0.83) | 3.5 (0.77) | 4.1 (0.55) | 0.8 (0.40) |
| Bodily Pain | 8.3* (0.77) | 3.5 (0.84) | 2.9 (0.71) | 1.2 (0.70) | 1.1* (0.62) | -1.5 (0.72) | 0.2 (0.50) | -0.7 (0.51) | 7.6* (1.24) | 2.0 (1.28) | 2.5* (1.26) | -2.4 (1.42) | 0.1 (0.88) | -1.4 (0.88) | -0.2 (0.85) | -2.6 (0.96) | 9.4 (1.16) | 5.9 (0.88) | 3.7 (0.80) | 0.0 (0.58) |
| Vitality | 7.6* (0.72) | 2.7 (0.79) | 2.8 (0.69) | 1.0 (0.68) | 2.7* (0.57) | -0.9 (0.65) | 0.7* (0.51) | -0.9 (0.54) | 5.0 (1.07) | 2.1 (1.11) | 1.5 (1.10) | -1.6 (1.24) | -0.7 (1.02) | -1.8 (1.02) | -0.6 (0.85) | -3.0 (0.95) | 9.3 (0.92) | 5.1 (0.78) | 3.7 (0.95) | 1.5 (0.64) |
| Social Functioning | 4.5* (0.67) | 2.1 (0.74) | 1.2 (0.56) | 0.1 (0.55) | 0.3* (0.50) | -1.4 (0.58) | -0.3 (0.37) | 0.4 (0.38) | 4.0 (0.91) | 1.8 (0.94) | 0.5 (1.04) | -1.9 (1.17) | -0.6 (0.79) | -1.2 (0.79) | -0.5 (0.63) | -2.0 (0.71) | 7.4 (0.98) | 1.6 (0.86) | 2.0 (0.64) | 0.4 (0.53) |
| Role-Emotional | 5.7* (0.81) | 2.3 (0.88) | 3.0* (0.64) | -0.1 (0.63) | 0.4 (0.56) | 0.5 (0.65) | -0.7 (0.40) | -0.4 (0.42) | 4.7* (1.22) | 0.9 (1.26) | 0.3 (0.91) | -0.8 (1.03) | -1.0 (0.99) | -1.6 (0.99) | -0.0* (0.85) | -3.1 (0.96) | 8.6 (1.16) | 2.4 (0.85) | 2.8 (0.71) | 0.9 (0.50) |
| Mental Health | 4.0* (0.71) | 1.7 (0.77) | 0.8 (0.68) | -0.5 (0.67) | 0.4 (0.60) | -0.8 (0.69) | -0.2 (0.49) | -0.4 (0.51) | 3.6 (1.06) | 1.2 (1.10) | 0.2* (1.08) | -3.3 (1.21) | -1.3 (0.99) | -0.9 (0.99) | -1.2 (0.88) | -2.3 (0.99) | 5.9 (0.98) | 2.6 (0.81) | 0.9 (1.02) | 1.0 (0.55) |
| **Results by IWQOL-Lite-CT Physical Function composite score quartiles at baseline** | | | | | | | | | | | | | | | | | | | | |
| **LSM (SE) change from baseline in SF-36 v2 domain scores** | | | | | | | | | | | | | | | | | | | | |
|  | SURMOUNT-1  TZP 15mg: N=627; PBO: N=639 | | | | | | | | SURMOUNT-3  TZP MTD: N=287; PBO: N=291 | | | | | | | | SURMOUNT-4 TZP MTD: N=335 | | | |
| Role-Physical | 6.7 (0.73) | 4.5 (0.88) | 4.3* (0.52) | 1.7 (0.50) | 1.4 (0.45) | 0.6 (0.47) | 0.2 (0.33) | -0.0 (0.35) | 6.0* (1.07) | 1.9 (1.03) | 1.6* (0.94) | -1.6 (0.98) | 0.7 (0.62) | -1.1 (0.66) | -0.1 (0.56) | -1.7 (0.64) | 8.3 (0.96) | 6.1 (0.71) | 3.0 (0.56) | 0.7 (0.33) |
| Bodily Pain | 7.6* (0.81) | 2.0 (0.96) | 2.4 (0.71) | 1.6 (0.69) | 2.1* (0.57) | -1.1 (0.60) | 0.3 (0.50) | -0.5 (0.53) | 5.9* (1.48) | 1.2 (1.42) | 4.2* (1.03) | -2.6 (1.08) | 0.9 (0.89) | 0.2 (0.95) | -0.4 (0.80) | -2.0 (0.92) | 9.6 (1.17) | 5.6 (0.88) | 3.0 (0.72) | 0.2 (0.62) |
| Vitality | 6.9* (0.76) | 2.6 (0.91) | 4.3* (0.67) | 0.4 (0.65) | 2.9* (0.53) | 0.2 (0.55) | -0.6 (0.55) | -1.4 (0.59) | 4.1 (1.22) | 1.2 (1.17) | 1.1 (1.24) | -2.0 (1.29) | 2.2* (0.84) | -1.3 (0.90) | -1.7 (0.79) | -2.0 (0.91) | 9.5 (0.97) | 5.2 (0.80) | 3.4 (0.72) | 1.3 (0.69) |
| Social Functioning | 3.7* (0.77) | 1.3 (0.92) | 1.7* (0.52) | -0.1 (0.51) | 0.7 (0.44) | 0.5 (0.46) | -0.3 (0.39) | -0.3 (0.42) | 3.8 (1.14) | 1.6 (1.10) | 0.0 (1.02) | -2.0 (1.07) | 0.4 (0.63) | -0.3 (0.67) | -0.6 (0.57) | -1.7 (0.66) | 6.5 (1.00) | 2.5 (0.85) | 1.7 (0.69) | 0.2 (0.51) |
| Role-Emotional | 4.6* (0.84) | 0.5 (1.01) | 2.5* (0.61) | 0.2 (0.59) | 0.8 (0.56) | 0.6 (0.59) | 0.1 (0.40) | -0.0 (0.42) | 4.3 (1.41) | 1.3 (1.35) | -0.7 (1.31) | -3.2 (1.37) | 0.6* (0.63) | -1.5 (0.67) | 0.1 (0.81) | -1.4 (0.93) | 6.6 (1.26) | 5.2 (0.75) | 1.9 (0.71) | 0.4 (0.48) |
| Mental Health | 2.1 (0.79) | 0.8 (0.94) | 2.1* (0.67) | -0.5 (0.65) | 0.5 (0.53) | -0.1 (0.55) | 0.1 (0.52) | -0.4 (0.56) | 2.9 (1.31) | 0.3 (1.26) | 0.6 (1.13) | -1.8 (1.18) | 0.3 (0.79) | -0.6 (0.85) | -1.8 (0.87) | -2.3 (1.00) | 5.1 (1.05) | 4.2 (0.81) | 0.5 (0.72) | 0.9 (0.65) |

*p<0.05 vs. placebo

Data are presented as LSM (SE) change from baseline (randomization [Week 0] for SURMOUNT-1 and -3, and lead-in baseline [Week 0] for SURMOUNT-4) at Week 72 (SURMOUNT-1 and -3) and Week 88 (SURMOUNT-4) using ANCOVA with LOCF.

In SURMOUNT-4, the comparator arm received both tirzepatide (weeks 0 – 36) and placebo (weeks 36 – 88). Thus, its results are not presented.

Abbreviations: ANCOVA, analysis of covariance; IWQOL-Lite-CT, Impact of Weight on Quality of Life-Lite-Clinical Trials Version; LOCF, last observation carried forward; LSM, least-squares mean; MTD, maximum tolerated dose; PBO, placebo; PRO, patient-reported outcome; Q1, first quartile; Q2, second quartile; Q3, third quartile; Q4, fourth quartile; SE, standard error; SF-36v2, Short Form-36 Version 2 Health Survey acute form; TZP, tirzepatide.

**Figure S1. Duration of obesity by baseline quartiles of IWQOL-Lite-CT Physical Function composite score.**


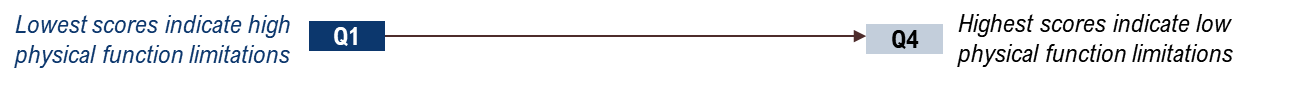


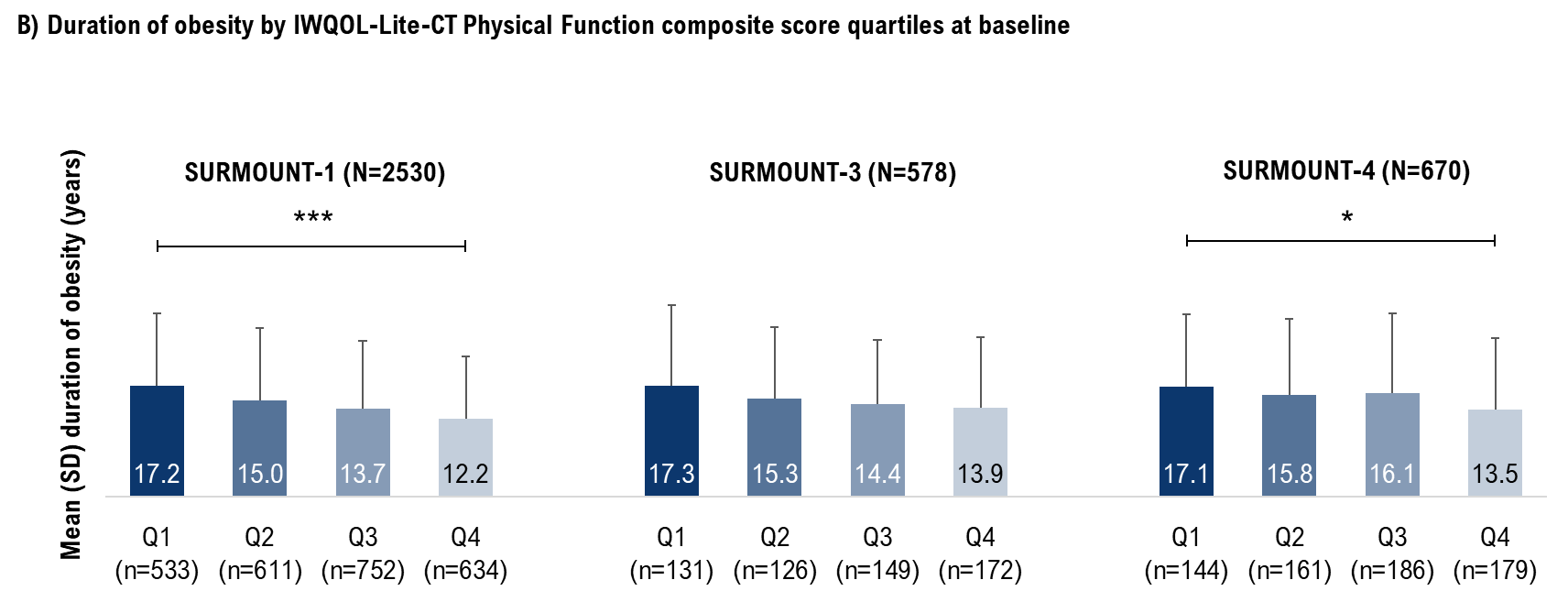


*p<0.05; ***p<0.001 across baseline quartiles based on ANOVA model.

Baseline is randomization (Week 0) for SURMOUNT-1 and SURMOUNT-3, and lead-in baseline (Week 0) for SURMOUNT-4.
Abbreviations: ANOVA, analysis of variance; IWQOL-Lite-CT, Impact of Weight on Quality of Life-Lite-Clinical Trials Version; Q1, first quartile; Q2, second quartile; Q3, third quartile; Q4, fourth quartile; SD, standard deviation.

**References**

1. Maruish ME. User’s Manual for the SF-36v2 Health Survey. 3rd ed. Lincoln, RI: Quality Metric Incorporated; 2011.

2. Kolotkin RL, Williams VSL, Ervin CM, et al. Validation of a new measure of quality of life in obesity trials: Impact of Weight on Quality of Life-Lite Clinical Trials Version. *Clin Obes*. Jun 2019;9(3):e12310. doi:10.1111/cob.12310

3. Kolotkin RL, Ervin CM, Meincke HH, Højbjerre L, Fehnel SE. Development of a clinical trials version of the Impact of Weight on Quality of Life-Lite questionnaire (IWQOL-Lite Clinical Trials Version): results from two qualitative studies. *Clin Obes*. Oct 2017;7(5):290-299. doi:10.1111/cob.12197
